# Genetic and gut microbiome determinants of SCFA circulating and fecal levels, postprandial responses and links to chronic and acute inflammation

**DOI:** 10.1080/19490976.2023.2240050

**Published:** 2023-08-01

**Authors:** Ana Nogal, Francesco Asnicar, Amrita Vijay, Afroditi Kouraki, Alessia Visconti, Panayiotis Louca, Kari Wong, Andrei-Florin Baleanu, Francesca Giordano, Jonathan Wolf, George Hadjigeorgiou, Richard Davies, Gregory A. Michelotti, Paul W. Franks, Sarah E. Berry, Mario Falchi, Adeel Ikram, Benjamin J. Ollivere, Amy Zheng, Jessica Nightingale, Massimo Mangino, Nicola Segata, William J. Bulsiewicz, Tim D. Spector, Ana M. Valdes, Cristina Menni

**Affiliations:** a Department of Twin Research, King’s College London, London, UK; b Department of Cellular, Computational and Integrative Biology, University of Trento, Trento, Italy; c Nottingham NIHR Biomedical Research Centre at the School of Medicine, University of Nottingham, Nottingham, UK; d Metabolon, Metabolon, Inc. Research Triangle Park, Morrisville, NC, USA; e Zoe Limited, London, UK; f Lund University Diabetes Center, Lund University, Malmö, Sweden; g Department of Clinical Sciences, Lund University, Malmö, Sweden; h Department of Nutritional Sciences, King’s College London, London, UK; i NIHR Biomedical Research Centre at Guy’s and St Thomas’ Foundation Trust, London, UK

**Keywords:** Short-chain fatty acids, postprandial, heritability, gut microbiota, inflammatory response

## Abstract

Short-chain fatty acids (SCFA) are involved in immune system and inflammatory responses. We comprehensively assessed the host genetic and gut microbial contribution to a panel of eight serum and stool SCFAs in two cohorts (TwinsUK, *n* = 2507; ZOE PREDICT-1, *n* = 328), examined their postprandial changes and explored their links with chronic and acute inflammatory responses in healthy individuals and trauma patients. We report low concordance between circulating and fecal SCFAs, significant postprandial changes in most circulating SCFAs, and a heritable genetic component (average *h^2^*: serum = 14%(SD = 14%); stool = 12%(SD = 6%)). Furthermore, we find that gut microbiome can accurately predict their fecal levels (AUC>0.71) while presenting weaker associations with serum. Finally, we report different correlation patterns with inflammatory markers depending on the type of inflammatory response (chronic or acute trauma). Our results illustrate the breadth of the physiological relevance of SCFAs on human inflammatory and metabolic responses highlighting the need for a deeper understanding of this important class of molecules.

## Introduction

Short-chain fatty acids (SCFA) are carboxylic acids formed by an aliphatic chain of 1–5 carbons^1^ mainly produced by colonic bacteria through the saccharolytic fermentation of resistant carbohydrates such as inulin, resistant starch and fructo-oligosaccharides, which escape digestion and absorption.^2^ The major formed SCFAs are acetate, propionate and butyrate, which account for approximately 80% of all SCFAs.^3^

Once produced, SCFAs can either be absorbed by the enterocytes or go into the bloodstream and reach different systemic tissues, exerting regulatory functions in gut barrier integrity, lipid and glucose metabolism, blood pressure, and immune function.^3^ Several studies have shown that SCFAs can also influence postprandial responses including postprandial glucose and insulin.^4,5^ For instance, a host-genetic-driven increase in gut production of butyrate was associated with improved postprandial insulin response.^4^

SCFAs can also exert anti-inflammatory effects, influencing chronic inflammation, by reducing the recruitment and migration of macrophages, dendritic cells, and neutrophils, and by altering T and B cell differentiation.^6^ Previously, levels of fecal SCFAs have been reported to be associated with mortality in critically ill patients with sepsis.^7,8^ However, the role of SCFAs in blood in acute inflammatory responses, such as those seen in acute trauma cases, has not been explored to date.

In human studies, SCFA levels have been associated both with gut bacterial species, including *Coprococcus*, *Bifidobacterium* and *Roseburia*,^3,9^ and with host genetics.^4^ Nonetheless, these studies do not simultaneously integrate SCFAs measured in both serum and stool, along with concurrent gut microbiome composition and genetic information. They also include only a subset of SCFAs available and focus on circulating fasting levels. Indeed, though humans spend most of their days in a postprandial state, postprandial SCFAs responses have only been investigated in animal models.^10,11^

By integrating data from a large population-based cohort and a postprandial interventional study, using healthy individuals we aimed to investigate (i) changes in SCFA levels after a meal challenge and (ii) the contribution of the host genetics and gut microbiome composition to their levels in serum and stool. We further aimed to understand the role of circulating SCFAs in chronic and acute inflammation by assessing their correlations with a set of pro- and anti-inflammatory markers in healthy individuals and in an acute fracture case-control study, as well as changes in their levels in response to acute inflammatory responses to trauma.

## Results

**Figure 1.**
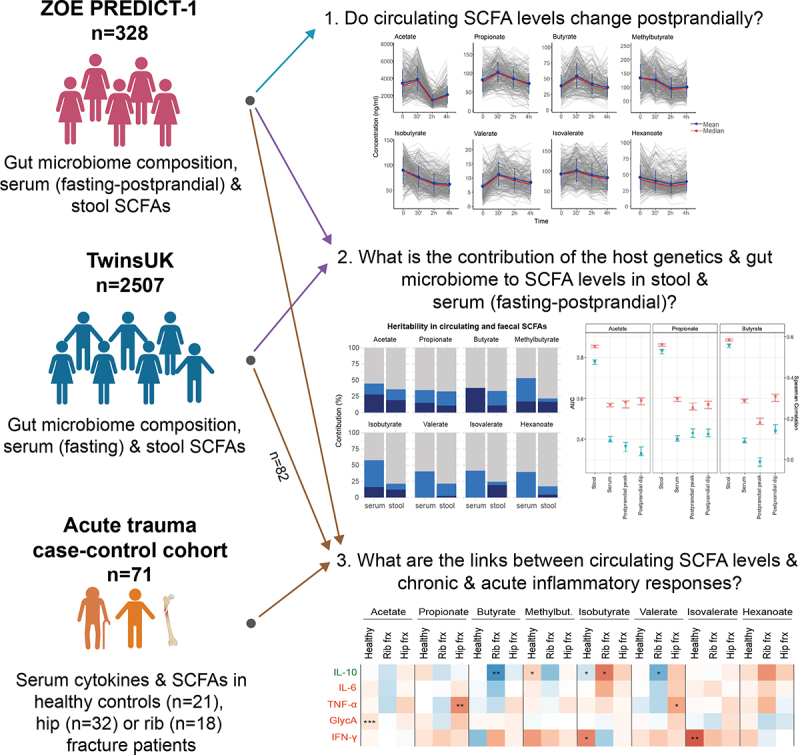
Flowchart of the study design. The three cohorts are independent. From each cohort, we included the subset of individuals who had short-chain fatty acids (SCFA) measured. TwinsUK and ZOE PREDICT-1 cohorts consist of healthy individuals, while the acute trauma case-control includes fracture patients and healthy individuals.

A flowchart of the study design is presented in Figure 1.

We included 2507 individuals (age mean = 57.9 ± 15.4 years, 2110 females) from the TwinsUK cohort and 328 females (age mean = 53.8 ± 7.1 years) from the ZOE PREDICT-1 cohort that had eight SCFAs, including acetate, propionate and butyrate, measured in stool and in fasting serum. Additionally, postprandial serum SCFAs were measured in ZOE PREDICT-1 participants. Both cohorts included twins. The demographic characteristics of the study populations and the mean baseline levels of the SCFAs in serum and stool are presented in Table 1.

**Table 1.** Demographic characteristics and SCFA levels of the study populations TwinsUK and ZOE PREDICT-1.

Study	TwinsUK		ZOE PREDICT-1	
n	2507		328	
Females, (%)	84%		100%	
Age, yrs	57.9 (15.4)		53.8 (7.1)	
BMI, kg/m^2^	25.87 (5)		26.24 (5.6)	
MZ:DZ:Singlet ons	1376:776:355		164:50:114	
SCFA	Fasting serum (ng/ml)	Stool (μg/g)	Fasting serum (ng/ml)	Stool (μg/g)
Acetate	3030 (1600)	4060 (1700)	3500 (1620)	4020 (1710)
Propionate	83.3 (29)	1320 (624)	84.9 (32.3)	1340 (676)
Butyrate	40.4 (19.5)	1420 (893)	41.4 (20.0)	1380 (924)
Isobutyrate	72.2 (24.3)	202 (87.7)	90.0 (27.1)	220 (88.8)
Methylbutyrate	117 (42.6)	157 (77)	134 (48.9)	170 (72.6)
Valerate	8.57 (4.47)	254 (119)	7.34 (4.76)	287 (129)
Isovalerate	78.7 (30)	193 (91.9)	93.2 (29.8)	212 (92.7)
Hexanoate	47.2 (17.2)	105 (114)	45.3 (20.1)	130 (121)

**Figure 2.**
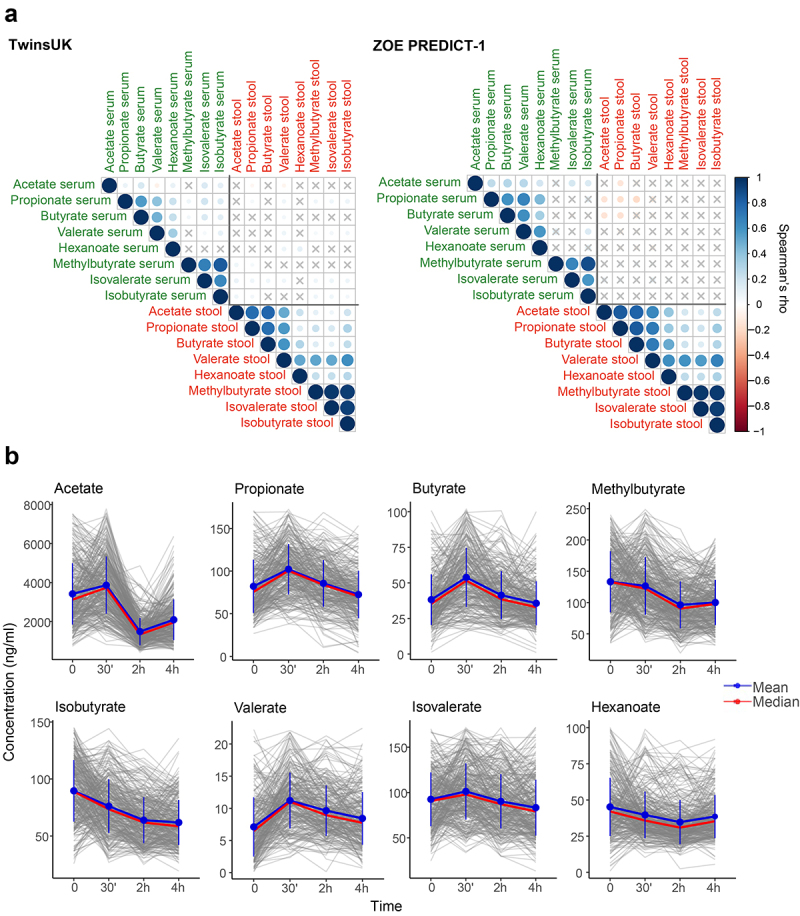
(a) Fasting circulating and faecal SCFAs correlation in TwinsUK (*n*=2229) and ZOE PREDICT-1 (*n*=328). Spearman’s correlations are presented. Non-significant correlations (FDR≥0.05) are indicated with a ‘X’. (b) Postprandial changes in circulating SCFA levels for 328 ZOE PREDICT-1 participants in response to a meal challenge under a controlled clinic setting at baseline and after 30 min, 2h and 4h. The bars indicate the standard deviations for each time point.

The correlations between circulating and fecal SCFAs calculated in TwinsUK and ZOE PREDICT-1 separately are presented in Figure 2a. We found non-significant or low correlations (ρ < ±0.15) between the circulating SCFAs with their respective fecal SCFAs in both studies. We also detected a low correlation between serum and fecal SCFAs and age and body mass index (BMI) (ρ < ±0.2) (Supplementary Figure S1).

### Postprandial changes in SCFA levels

As individuals spend most of the day in a postprandial state (i.e., not fasting), we investigated whether circulating SCFA levels change after a standardized meal and their inter-individual variability. For that, we took advantage of the ZOE PREDICT-1 cohort, in which SCFA levels were measured in a tightly controlled clinic setting at fasting, 30 min, 2 h, and 4 h after a meal challenge. Each participant consumed a standardized muffin, as described in the Methods section, that included on average 2.25 g of dietary fiber (50 g fat and 85 g carbohydrate). The changes in the SCFA levels in response to it are depicted in Figure 2b. We report a significant postprandial change in all SCFAs from fasting (Wilcoxon test, p < 0.05) except for peak hexanoate and butyrate, and dip valerate (Supplementary Table S1). Compared to the fasting measure, SCFA levels changed on average by at least 1.03-fold for methylbutyrate and by as much as 2.6 folds for acetate. The coefficient of variation (CV) indicated a moderate postprandial inter-individual variability in their highest (CV range = 26.5–39.8%) and lowest concentrations (CV range = 32–46.7%) (Supplementary Table S1). We found only weak non-statistically significant associations between postprandial SCFAs and postprandial lipemic and glycemic parameters (2-h glucose iAUC, rise in triglyceride at 6 h postprandially, rise in insulin at 2 h postprandially and rise in C-peptide at 2 h postprandially), with the exception of the significant associations with the rise of triglycerides at 6 h postprandially with dip acetate, and 2-h glucose iAUC with dip valerate and acetate (Supplementary Table S2).

### Host genetics contribution to SCFA levels

**Figure 3.**
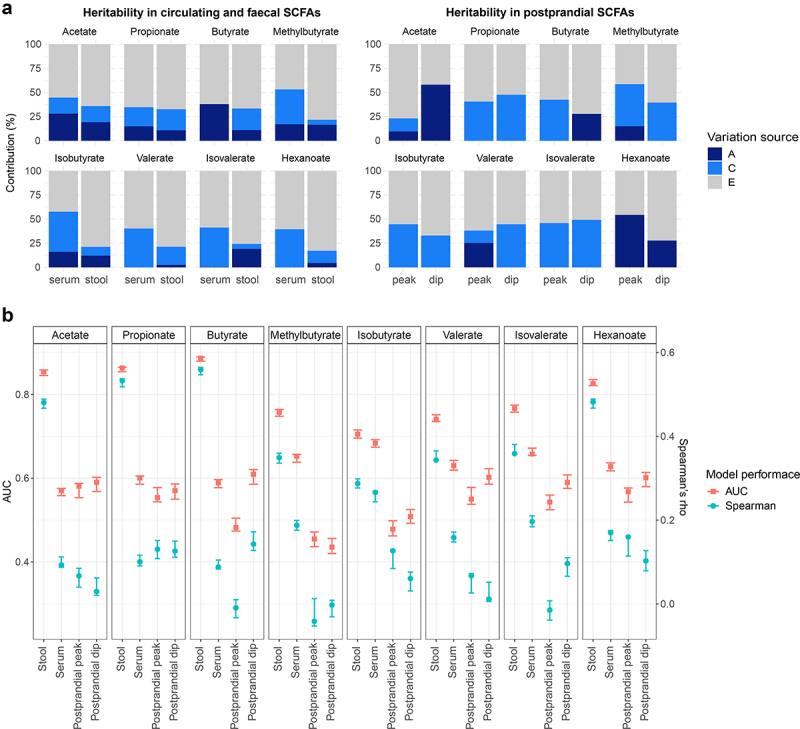
Contribution of host genetics and gut microbiome composition to SCFA levels in serum and stool. Analyses using serum at fasting and stool measurements were performed using the TwinsUK and ZOE PREDICT-1 participants together, while analyses using postprandial measurements were run using ZOE PREDICT-1 participants. Postprandial measures were defined as peak (the maximum SCFA concentration in the 4 hours following the test meal challenge minus the fasting level) and dip (the fasting level minus the minimum SCFA concentration in the 4 hours following the test meal challenge) (a) Heritability estimates of (left) fasting circulating and fecal SCFAs, and (right) postprandial circulating SCFAs. A, C and E labeling indicates the amount of variance attributed to the additive genetic factors or heritability, common/shared environmental factors, and unique environmental factors/error, respectively. (b) Influence of the gut microbiota composition in fecal and circulating (fasting and postprandial) SCFA levels estimated by Random Forest regression (using Spearman’s correlations) and classification (using AUC) models. Blue bars indicate the median and the 95% confidence intervals of the correlation between the real value of each component and the value predicted by regression models across 100 training/testing folds. Red bars represent the median AUC and the 95% confidence intervals across 100 folds for a corresponding binary classifier between the highest and lowest quartile.

We estimated the contribution of host genetics to the SCFA levels in serum and stool by calculating heritability estimates using structural equation modeling adjusted for covariates and pooling together TwinsUK and ZOE PREDICT-1 participants (serum: *n* = 2835, 1540 monozygotic (MZ) pairs, 826 dizygotic (DZ) pairs, and 469 singletons; stool: *n* = 2557, 1258 MZ pairs, 734 DZ pairs, and 565 singletons). The estimated additive genetic component (*h*^*2*^) for circulating SCFA levels were on average 14% (SD = 14%), ranging from 0% for valerate (95%CI = 0%,0%), isovalerate (95%CI = 0%,19%), and hexanoate (95%CI = 0%,0%), to 38% (95%CI = 32%,44%) for butyrate. In stool, the estimated additive genetic component was on average 12% (SD = 6%) ranging from 3% (95%CI = 0%,27%) for valerate to 19% for acetate (95%CI = 0%,42%) and isovalerate (95%CI = 0%,44%) (Figure 3a). Acetate, propionate and butyrate presented larger heritability estimates for serum (average *h*^*2*^ = 27%(SD = 12%)) than for stool levels (average *h*^*2*^ = 14%(SD = 5%)). In a sub-analysis, we also explored the host genetics contribution to the postprandial SCFA levels in ZOE PREDICT-1 participants (*n* = 328, 164 MZ pairs, 50 DZ pairs and 114 singletons). The ACE model obtained for the peak and dip calculated for each SCFA revealed that postprandial levels of propionate, isobutyrate and isovalerate are environmentally driven, whereas hexanoate and acetate have a large genetic component with heritability estimates of 54% (95%CI = 37%,72%) and 10% (95%CI = 0%,82%) for the peak, and of 28% (95%CI = 4%,52%) and 58% (95%CI = 43%,73%) for the dip, respectively (Figure 3a).

### Gut microbiota contribution to SCFA levels

As SCFAs are gut microbial-derived metabolites, we investigated the gut microbiome contribution to SCFA levels in serum and stool using RF classifiers and regressors trained on relative abundance values of gut microbiome species. The performance was evaluated with the median AUC values for the classifiers and the median Spearman’s correlation values (defined as “ρ”) for the regressors over 100 bootstrap folds (see Methods). We found that the gut microbiota was able to accurately predict the fecal SCFA levels (AUC>0.71 and ρ>0.29), with the strongest associations observed for acetate: AUC[95%CI] = 0.85 [0.85,0.86], ρ[95%CI] = 0.48 [0.47,0.49]; propionate: AUC[95%CI] = 0.86 [0.85,0.87], ρ[95%CI] = 0.53 [0.52,0.54] and butyrate: AUC[95%CI] = 0.89 [0.88, 0.89], ρ[95%CI] = 0.56 [0.55,0.56] (Figure 3b). We also identified *Akkermansia muciniphila*, *Faecalibacterium prausnitzii* and *Roseburia* spp., among others, as important predictors (Supplementary Figure S2). On the other hand, a moderate association was found between the gut microbiota and circulating SCFAs with an AUC and ρ average of 0.63 (95%CI = 0.61,0.63) and 0.15 (95%CI = 0.14,0.16), respectively (Figure 3b). These findings were consistent with the results obtained for TwinsUK and ZOE PREDICT-1 separately (Supplementary Table S3). In a sub-analysis, we also report that postprandial SCFA levels are poorly linked with gut microbiome composition (average AUC = 0.53 (95%CI = 0.51,0.54) and ρ =0 .07(95%CI = 0.05,0.08) for the peak, and average AUC = 0.56 (95%CI = 0.54,0.58) and ρ = 0.07(95%CI = 0.05,0.09) for the dip (Figure 3b).

### Circulating SCFA levels in chronic and acute inflammation

SCFAs are known to modulate immune responses by regulating the production of immune cells and cytokines ^6^. We thus investigated the role of circulating SCFAs in chronic and acute inflammatory responses. A deep understanding of how SCFAs can influence inflammation is crucial for developing strategies for the management and recovery of patients with inflammatory disorders.

**Figure 4.**
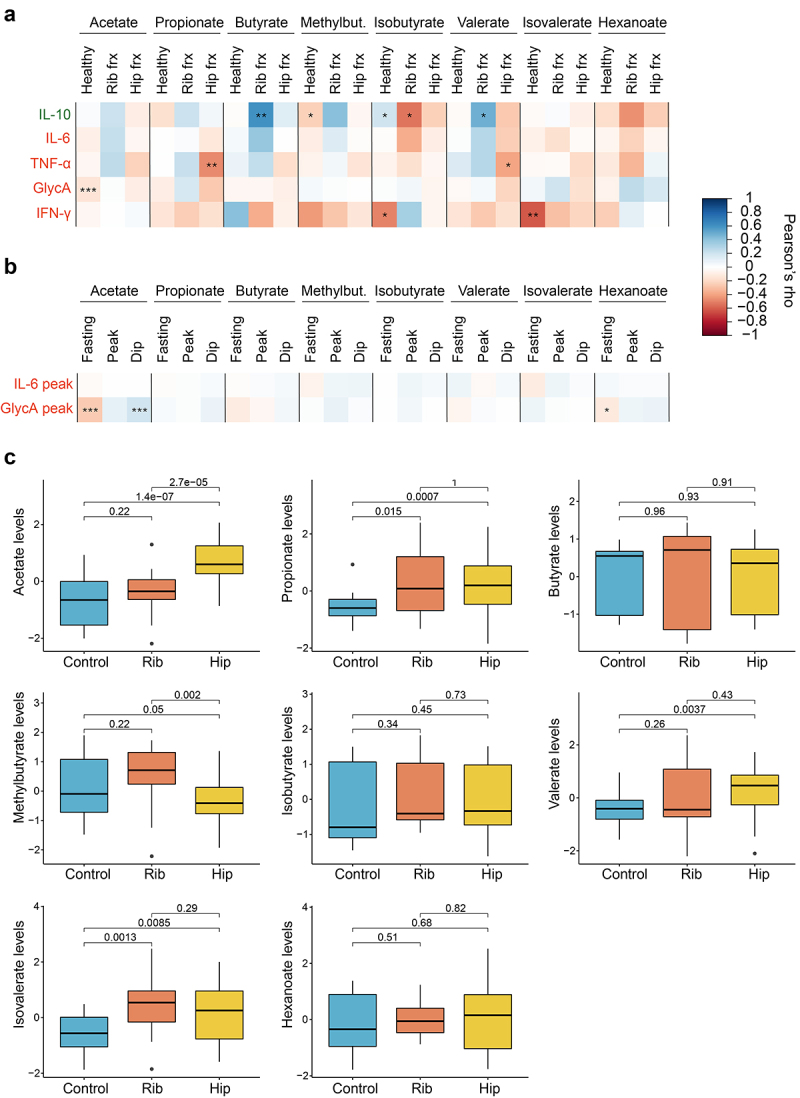
Role of circulating SCFAs in chronic and acute inflammation. (a) Pearson’s correlations between SCFA levels and anti- (IL-10) and pro-inflammatory (IL-6, TNF-α, GlycA,IFN-γ) markers stratified by healthy individuals and acute trauma cases. For the healthy group, correlation results obtained in the healthy individuals from the acute trauma case-control cohort, in the subset from TwinsUK and ZOE PREDICT-1 were combined by applying inverse variance random effect meta-analysis. Cases are from the acute trauma case-control cohort. The controls illustrate the links between SCFAs and chronic inflammation, whereas the cases show the links between SCFAs and acute inflammatory responses. (b) Pearson’s correlations between fasting and postprandial SCFA levels and the postprandial pro-inflammatory markers available in ZOE PREDICT-1 (IL-6 and GlycA). (c) Differences in the SCFA levels between controls and cases in the acute trauma case-control cohort. The p-values obtained from t-tests between groups are indicated. For these analyses, only individuals with both serum SCFAs and cytokines are included (i.e., TwinsUK, n=82; ZOE PREDICT-1, n= 328; acute trauma case-control cohort: controls, n= 21, rib fracture, n= 18, hip fracture, n=32). Levels were log-transformed and Z-scaled. P-value: *0.05; **0.01; ***0.001. Pro- and anti-inflammatory cytokines are colour-coded in red and green, respectively. Abbreviations: Frx: Fracture; IFN, interferon; IL, interleukin; Methylbut., methylbutyrate; TNF, tumour necrosis factor.

We first investigated the relationship between circulating SCFA levels and systemic inflammation. For that, we performed Pearson’s correlations between serum levels of SCFAs and cytokines in the 328 individuals from ZOE PREDICT-1, a subset of 82 women from TwinsUK, and in 21 healthy individuals from the acute trauma case-control study (see Supplementary Table S4 for descriptive characteristics). We then combined the results from the different studies using an inverse variance random effect meta-analysis (Figure 4a). We found that healthy individuals tended to have negative correlations with pro-inflammatory markers including interferon-gamma (IFN-γ) (isovalerate: ρ=-0.61, p-value = 0.004; isobutyrate: ρ=-0.5, p-value = 0.03) and GlycA (acetate: ρ=-0.14, p-value = 0.0005).

We further explored whether there were any links between fasting and postprandial changes in SCFA and postprandial interleukin-6 (IL-6) and GlycA levels ^12^ in the ZOE PREDICT-1 participants (Figure 4b). We found postprandial GlycA (measured as the highest concentration within 6 h postprandially) to be strongly correlated with fasting (ρ=-0.26, *p*-value = 9.2 × 10^–6^) and postprandial (ρ = 0.2, p-value = 0.0008) acetate, while no significant correlations were found with postprandial IL-6.

We then investigated whether there were links between serum SCFAs and acute inflammatory responses measuring SCFA levels and their correlations to inflammatory markers in serum samples taken immediately preoperatively from individuals who had undergone either fragility hip fractures (*n* = 32) or multiple rib fractures (*n* = 18) requiring surgery (see Supplementary Table S4 for descriptive characteristics). The fragility fractures are measured in individuals with frailty (i.e., with high systemic inflammation)^13^ whereas the rib fractures cases are individuals who were otherwise healthy before the trauma.

When we assessed SCFA-cytokines correlations in the acute trauma cases (Figure 4a), we identified different patterns depending on whether the individual had a rib or a hip fracture. Specifically, the hip fracture patients presented significant negative correlations between the pro-inflammatory marker tumor necrosis alpha (TNF-α) and two SCFAs, namely propionate (ρ=-0.47, p-value = 0.007) and valerate (ρ=-0.39, p-value = 0.03). On the other hand, patients with multiple rib fractures presented significant associations with the interleukin-10 (IL-10), either positive or negative. Butyrate and valerate were positively associated with IL-10 levels (butyrate: ρ = 0.6, p-value = 0.007; valerate: ρ = 0.48, p-value = 0.04), whereas isobutyrate was presenting the opposing direction (ρ=-0.54, p-value = 0.02). No significant associations were found with IL-6 levels. Likewise, when comparing the circulating SCFA levels between healthy controls, rib or hip fracture patients using pairwise t.tests, we observed that acetate levels were significantly different in the three groups, whereas propionate and isovalerate levels in trauma cases were significantly higher than in the controls, and valerate levels were higher in the hip fracture patients in comparison with the controls. On the other hand, patients with a hip fracture presented significantly lower levels than patients with multiple rib fractures (Figure 4c). Results were consistent when running linear models and when further adjusting for age and sex (Supplementary Table S5).

## Discussion

To our knowledge, this is the first study to date investigating simultaneously the contribution of host genetics and gut microbiome to the fasting and postprandial levels of eight SCFAs in serum and stool in two independent cohorts of healthy individuals. Specifically, we have shown that (i) there is a very low concordance between fecal and circulating SCFA levels, which might be due to the fact that most absorbed SCFAs act as an energy source for the enterocytes and are not systemically transported^3^, (ii) SCFA levels change postprandially and there are substantial inter-individual differences in these responses, (iii) stool and serum SCFA levels are heritable, with the exception of circulating valerate, isovalerate and hexanoate that are environmentally determined, (iv) most postprandial SCFA levels appear to be environmentally driven, (v) the gut microbiome composition is an important contributor of fecal levels, but presents weaker associations with circulating levels. Importantly, using an independent acute trauma case-control cohort, we report for the first time that circulating SCFA levels vary between trauma patients and controls and that there is a different relationship between pro- and anti-inflammatory cytokines and SCFAs depending on the type of inflammatory response (chronic or acute).

We found that a large proportion of the SCFA levels in serum and stool are explained by environmental factors, which is in line with the proposed importance of non-genetic factors in the SCFAs formation, including the different environmental factors modulating the gut microbiota.^14^ Of note, we found that the three most widely studied SCFAs (acetate, butyrate and propionate) had moderate heritability estimates, with a larger genetic contribution to serum (average *h*^*2*^ = 27%(SD = 12%)) than to stool levels (average *h*^*2*^ = 14%(SD = 5%)). Our results are supportive of Sanna and coworkers previous report suggesting that host genetics influence the microbial expression of propionate and butyrate.^4^ The lower heritability for stool level we found is not surprising given that fecal levels are more likely to reflect SCFA bacterial generation, whereas serum levels will reflect absorption from the gut but also synthesis by the host (e.g., acetate is a metabolite involved in the tricarboxylic acid (TCA) cycle).^15^ On the other hand, we were unable to detect a genetic contribution for the postprandial levels of most SCFAs, similar to what we observed in this same cohort for postprandial c-peptide and insulin responses (see^16^). The only exceptions were postprandial acetate and hexanoate both of which presented large heritability estimates, which can be due to the fact that, as previously mentioned, acetate is involved in the TCA cycle,^15^ and hexanoate can be also generated by hepatic peroxisomal beta-oxidation of long-chain fatty acids.^17^

When investigating the contribution of the gut microbiome, we found that it can accurately predict SCFA levels in stool (AUC>0.71), however, the predictive power is reduced for serum levels, both fasting and postprandial. This is consistent with our findings indicating that SCFA levels in serum and stool are not correlated with each other. These observations highlight the fact that fecal levels are not representative of the actual absorption and suggest that caution should be taken when inferring microbiome-disease associations,^18^ from either serum or fecal SCFA levels. Both types of measurements are needed to fully understand the role of SCFAs in health. Moreover, we were able to identify the key gut microbial species modulating SCFAs fecal abundances. These include the widely known SCFA producers *F. prausnitzii*,^19^ *Roseburia* spp,^20^ or *C. comes*,^9^ among others, positively correlated with acetate, propionate and butyrate, which also confirms the robustness of our methodology. On the other hand, we identify *Alistipes* spp. showing negative associations. *Alistipes* spp. has been associated with gut dysbiosis and chronic inflammation diseases.^21^ Of note, some of the identified species were showing an opposing direction between acetate, propionate and butyrate, and isobutyrate, methylbutyrate and isovalerate (e.g., *R. lactatiformans* is negative for the first three and positive for the last three). This can be explained by the distinct substrate used to produce SCFAs.^22^ Indeed, acetate, butyrate and propionate are mainly produced by the fermentation of resistant carbohydrates,^23^ while isobutyrate, methylbutyrate and isovalerate are mainly produced from the amino acid fermentation.^24^

We also explored the links between serum SCFAs and chronic and acute inflammation. When examining the correlations between cytokines and SCFA levels in healthy individuals, we observed that SCFAs are linked to lower systemic inflammation consistent with these compounds being involved in downregulating pro-inflammatory markers (e.g., isovalerate and isobutyrate vs IFN-γ) and their postprandial responses (e.g., fasting and postprandial acetate vs postprandial GlycA). This is in line with the already reported benefits of SCFAs in chronic inflammation.^25^ In acute trauma patients we report that hip fragility fractures and multiple rib impact fractures led to differences in some circulating SCFA levels with respect to healthy individuals and with each other. According to animal studies, SCFAs are mostly metabolized by the muscles and kidneys.^11^ After a fragility fracture, like hip fractures, acute kidney injury is a frequent complication, whereas it is significantly less frequent in impact fractures, like rib fractures,^26^ which might explain some of the observed differences in SCFA levels between distinct trauma patients and healthy individuals. When we analyzed the observed differences in the acute trauma patients in relation to their correlations with inflammatory markers, we noted that SCFA levels were positively correlated with the anti-inflammatory cytokine IL-10 (butyrate and isovalerate) but only among rib fracture, whereas SCFA levels were negatively associated with pro-inflammatory cytokines in hip fracture cases (e.g., TNF-α vs propionate and valerate). Therefore, the associations between SCFAs and cytokines are different in rib fractures (young and healthy before trauma) and hip fractures (frail elderly individuals). Importantly, mortality rates within 30 days of fractures are < 1% for rib fractures for individuals under the age of 65^27^ and > 10% for fragility hip fractures^28^ which is also one of the global top 10 causes of disability in adults.^29^ The differences in inflammatory responses in these two trauma scenarios suggest that SCFAs and their links to pro- and anti-inflammatory pathways might be related also to the recovery process. Taken together, SCFAs might help to dampen the inflammatory response in acute inflammation, while they might contribute to the maintenance of a low-grade inflammatory state in systemic inflammation by influencing fasting and postprandial inflammation.

We acknowledge the following study limitations. First, in ZOE PREDICT-1, SCFAs were measured only in women, and therefore, postprandial results might not be generalized to men. Unfortunately, we could not compare the postprandial findings in TwinsUK, which include men and women, as postprandial measurements are not available for this cohort. Likewise, it was not possible to assess the postprandial responses in the acute trauma case-control cohort as samples were collected in an acute hospital setting. The heritability analyses that exclusively include the ZOE PREDICT-1 participants lack statistical power, and the results might differ if more participants are included. Lack of power also prevented us from examining the genetic factors that influence postprandial levels of SCFAs. Although we meta-analyzed the correlations obtained from the acute trauma case-control, a subset of TwinsUK and ZOE PREDICT-1 studies, not all of them presented the same cytokines measures. Besides, postprandial levels were only available for GlycA and IL-6 in the ZOE PREDICT-1 study, and the data used in this study does not allow us to infer causality between SCFA levels and the studied inflammatory markers. Finally, we were unable to evaluate variations in SCFA levels over time as longitudinal SCFA data was unavailable for the included cohorts.

Despite the above limitations, we benefit from an independent and well-characterized large population-based study, a detailed postprandial interventional study and an acute fracture case-control study that allowed us to investigate the link between circulating SCFAs and acute inflammation.

In conclusion, in the most comprehensive study to date examining the contribution of host genetics and gut microbiome composition to fecal and circulating levels in two independent population-based cohorts, our findings indicate that SCFA levels are mostly modifiable and change postprandially, and fecal SCFAs reflect the gut microbiome composition. We also show for the first time that the SCFA profile and their correlations with inflammatory markers change depending on the type of inflammatory response (chronic or acute trauma). Taken together, our results illustrate the breadth of the physiological relevance of SCFAs on human inflammatory and metabolic responses highlighting the need for a deeper understanding of this important class of molecules.

## Patients and methods

### Study populations

This study consists of three completely independent cohorts. TwinsUK and ZOE PREDICT-1 consist of healthy individuals, whereas the acute trauma case-control cohort includes three subsets of individuals (healthy individuals, patients with rib fractures and patients with hip fractures). The acute trauma case-control cohort was included to exemplify the role of circulating SCFA levels in an acute inflammatory situation, as the rest of the work focused on SCFA levels in healthy individuals.

#### TwinsUK

TwinsUK registry is a national register of adult twins recruited as volunteers without selecting for any particular disease or trait.^30^ We included 2507 and 2229 individuals with serum and fecal SCFA measurements, respectively. For those, 2197 had measurements in both stool and serum. Along with the SCFA measurements, shotgun metagenomes from the gut microbiome were also available. A subset of 82 individuals also had measurements of circulating cytokines. The study was approved by NRES Committee London – Westminster, and all twins provided informed written consent.

#### ZOE PREDICT-1

We included a subset of 328 individuals from the UK-based ZOE PREDICT-1 study with SCFAs measured in serum and stool, and gut microbiome composition assessed with shotgun metagenomes. The ZOE PREDICT-1 study^16^ was a single-arm nutritional intervention conducted between June 2018 and May 2019. Study participants were healthy individuals (thus eliminating potential confounders brought about by the presence of infections or other comorbidities) aged between 18–65 years recruited from the TwinsUK registry,^30^ and the general population using online advertising. Although the ZOE PREDICT- 1 participants were recruited from the TwinsUK registry, in this study the two cohorts, ZOE PREDICT-1 and TwinsUK, are completely independent and there is no overlap in participants. Participants attended a full-day clinical visit consisting of test meal challenges followed by a 13-day home-based phase, as previously described.^16^

##### Test meal challenge

Within a tightly controlled clinical setting, participants consumed meal 1: breakfast muffins and a milkshake (890 kcal, 85.5 g carbohydrate (38.4%), 52.7 g fat (53.3%), 16.1 g protein (7.2%), and 2.3 g fiber at the 0-hour timepoint, following baseline blood draw). Venous blood samples were collected at 15, 30, 60, 120, 180, 240, 300, 360 minutes post-meal 1.

#### Acute trauma case-control cohort

Patients were all recruited at Queens Medical Hospital part of the Nottingham University Hospital’s (NUH) NHS Trust.

##### Rib fracture cohort (OPERA)

Inclusion criteria were: adult patients (16 years and above) presenting multiple (3+) rib fractures suitable for surgical repair and having, as per British Orthopaedic Association Audit Standards For Trauma (BOAST-15) Standard 8, indications for fixation as: clinical flail chest; respiratory difficulty requiring respiratory support or uncontrollable pain using standard modalities; was a surgical candidate.

Patients were excluded if: they had a head or thoracic injury requiring emergency intervention; could not be operated on within 72 hours as unfit for surgery; presented with significant thoracic injury requiring surgery where conservative management would be inappropriate. Blood samples were taken at the time of the patient going into anesthesia ahead of entering the operating theater.

##### Hip fracture cohort (FEMUR)

Inclusion criteria: age over 65 (no upper age limit), Rockwood frailty score ≥ 4, fractured hip sustained following a fall that required surgery. Good understanding of spoken and written English language, ability to give informed consent or to provide assent and availability of a legally acceptable surrogate to provide consent. Exclusion criteria: those who fell and sustained the hip fracture more than 12 hours prior to hospitalization. Patients who had fallen and sustained a hip fracture whilst in-patient. Surgery that had to be delayed to 96 hours or more after the fall.

##### Control cohort with SCFAs and cytokines

Healthy students from the School of Medicine at the University of Nottingham or healthcare workers.

#### Ethics

TwinsUK: This study was carried out under TwinsUK BioBank ethics, approved by North West – Liverpool Central Research Ethics Committee (REC reference 19/NW/0187), IRAS ID 258,513. This approval supersedes earlier approvals granted to TwinsUK by the St Thomas’ Hospital Research Ethics Committee, later London – Westminster Research Ethics Committee (REC reference EC04/015), which have now been subsumed within the TwinsUK BioBank.

ZOE PREDICT-1: The study was approved by the London – Hampstead Research Ethics Committee (REC reference 18/LO/0663) and the trial was registered on ClinicalTrials.gov (registration number: NCT03479866).

The rib fractures cohort was collected as part of The Operative Rib Fixation (ORiF) Study (REC Reference: 18/SC/066, IRAS 248,460, IRSCTN 10,777,575). The hip fracture cohort was collected under Functioning of Elder Muscle; Understanding Recovery (FEMUR) study (REC approval: 20/LO/0841 clinicaltrials.gov registration NCT04764617). The control individuals collected alongside were collected under REC ref FMHS 302–0621 by the internal review board by the University of Nottingham School of Medicine.

All participants provided written informed consent.

### SCFA measurements

Metabolomic profiling was performed on 2906 serum samples (2507, 328 and 71 participants from TwinsUK, ZOE PREDICT-1 and the acute trauma case-control cohort, respectively) and 2557 stool samples (2229 and 328 participants from TwinsUK and ZOE PREDICT-1, respectively) by Metabolon Inc. using liquid chromatography coupled with tandem mass spectrometry (LC-MS/MS), as previously described.^31^ For TwinsUK and ZOE PREDICT-1 cohorts, stool and fasting serum samples were available, whereas only fasting serum samples were available for the acute trauma case-control cohort, as samples were collected in a hospital setting. For the ZOE PREDICT-1 participants, postprandial (30 min, 2 h and 4 h) serum samples were also collected after consuming a standardized meal (see Methods:Test meal challenge). Full details and quality control are included in Supplementary Text 1. In all the samples, the SCFA acetate, propionate, butyrate, methylbutyrate, isobutyrate, valerate and isovalerate, and the medium-chain fatty acid hexanoate were measured. For the sake of ease of reading, hexanoate is included in the definition of SCFA.

### Postprandial metrics

For each SCFA, we defined as (i) peak the maximum SCFA concentration in the 4 hours following the test meal challenge minus the fasting level, and (ii) dip the fasting level minus the minimum SCFA concentration in the 4 hours following the test meal challenge. Postprandial lipemic and glycemic parameters (the 2-h glucose iAUC, rise in triglyceride at 6 h postprandially, rise in insulin at 2 h postprandially and rise in C-peptide at 2 h postprandially - see Berry et al., 2020^16^ for more details), and cytokines (the highest concentration of GlycA and IL-6 within 6 h postprandially) were also available.

### Microbiome sequencing and profiling

Deep shotgun metagenomic sequencing in stool samples from TwinsUK and ZOE PREDICT-1 was performed as previously described,^22,32^, and as detailed below.

#### Faecal sample collection

TwinsUK and ZOE PREDICT-1 participants collected stool samples at home in pre-labeled kits (containing 2 × 25 ml tube or 1 × 25 ml tube and 1 × 10 ml Zymo buffer) posted to them prior to their clinic visit date and brought with them to the visit. Alternatively, samples can be posted to the clinic using blue Royal Mail safe boxes. In the laboratory, samples were homogenized, aliquoted into 4 bijou tubes, and stored at − 80°C, within 2 hours of receipt.

#### DNA extraction, library preparation and DNA sequencing

To isolate genomic DNA from fecal material in TwinsUK, bijou tubes are removed from the freezer and ground with glass beads and 5-6 ml distilled water (Spex Grinder, 10 seconds, 800 strokes per minute). The supernatant is centrifuged and ground further (5 minutes, 1000 strokes per minute) before 200–300 µl of the sample is mixed with 10 µl PK solution and 720 µl of Lysis/Bind Master Mix). Proteins are degraded by the binding solution and subsequently extracted by KingFisher Flex robot. DNA is washed in 2 steps by washing solutions and eluted in MagMax Core Elution Buffer in 100 µl. In ZOE PREDICT-1, DNA was isolated by QIAGEN Genomic Services using DNeasy 96 PowerSoil Pro from the microbiome samples. Library preparation and sequencing were performed by GenomeScan for TwinsUK. For ZOE PREDICT-1, the quality and yield after sample preparation were measured with the Fragment Analyzer system following the manufacturer’s guidelines. The size of the resulting product was consistent with the expected size of approximately 500–700 bp. Libraries were sequenced for 300 bp paired-end reads using the Illumina NovaSeq6000 platform according to the manufacturer’s protocols. 1.1 nM library was used for flow cell loading. NovaSeq control software NCS v1.5 was used. Image analysis, base calling, and the quality check were performed with the Illumina data analysis pipeline RTA3.3.5 and Bcl2fastq v2.20.

#### Metagenome quality control and preprocessing

TwinsUK sequenced metagenomes were processed using the YAMP pipeline (v. 0.9.5.3).^33^ Briefly, identical reads were removed. Reads were filtered to remove adapters, known artifacts, phi X 174, and then quality trimmed (PhRED quality score < 10). Reads that became too short after trimming (*N* < 60 bp) were discarded. We retained singleton reads (i.e., reads whose mate has been discarded) to retain as much information as possible. Contaminant reads belonging to the host genome were removed (build: GRCh37). Low-quality samples, i.e., samples with <10 M reads after QC were discarded (*n* = 4). Sequenced metagenomes in ZOE PREDICT-1 were QCed using the pipeline implemented in https://github.com/SegataLab/preprocessing.

### Microbiome taxonomic profiling

The metagenomic analysis was conducted following the general guidelines^34^ and based on the bioBakery computational environment.^35,36^ High-resolution taxonomic profiling of the TwinsUK and ZOE PREDICT-1 metagenomes was performed using MetaPhlAn 4.beta.2 with the Jan21 database that comprises 26,970 species-level genome bins, with default parameters.^37^

### Inflammatory markers measurements

Pro-inflammatory markers TNF-α, IFN-γ, GlycA and IL-6, and the anti-inflammatory marker IL-10 were measured by ELISA by Affinity biomarkers in the acute trauma case-control cohort. In TwinsUK, IL-10, TNF-α, and IL-6 were measured using the bead-based high-sensitivity human cytokine kit (HSCYTO-60SK, Linco-Millipore) according to the manufacturer’s instructions. In ZOE PREDICT-1, IL-6 was measured by Affinity Biomarkers Lab using a Sandwich Immunoassay by Meso Scale Diagnostics. In TwinsUK and ZOE PREDICT-1, GlycA was measured using the high-throughput NMR metabolomic (Nightingale) 2016 panel.

**Data availability statement** The data used in this study are held by the Department of Twin Research at King’s College London. The data can be released to bona fide researchers using our normal procedures overseen by the Wellcome Trust and its guidelines as part of our core funding (https://twinsuk.ac.uk/resources-for-researchers/access-our-data/). The gut microbiome data is available on EBI (https://www.ebi.ac.uk/) under accession numbers PRJEB39223 (ZOE- PREDICT-1) and PRJEB32731 (TwinsUK).

### Statistical analyses

Statistical analyses were performed using RStudio version 1.3.1093, and python. All the models were corrected for multiple testing using false discovery rate (FDR – Benjamini and Hochberg method).^38^ If not indicated otherwise, the level of statistical significance was set at FDR < 0.05 in all the analyses. Before running the analyses, outliers of SCFA measures defined as values 4 standard deviations from the mean were excluded, and values were Z-scaled. Analyses with postprandial SCFA levels were performed only in ZOE PREDICT-1. To achieve the second aim of this study, TwinsUK cohort and ZOE PREDICT-1 were processed together, and findings were checked for consistency with results obtained for each individual cohort. To achieve the last aim, data from ZOE PREDICT-1, a subset of TwinsUK, and the acute trauma case-control cohort was included (see Figure 1).

#### Correlations between circulating and fecal SCFA levels

To investigate the correlations between circulating and fecal SCFA levels, we used non-parametric Spearman’s correlations as the SCFA measurements in TwinsUK and ZOE PREDICT-1 did not follow a normal distribution.

### Changes in postprandial SCFA levels and associations with postprandial lipemic and glycemic parameters

We examined postprandial changes from fasting in each SCFA using the Wilcoxon test, and the inter-individual variability in the highest and lowest postprandial concentration of each SCFA using the coefficient of variation (CV – calculated as SD/mean, %). We assessed the associations with postprandial SCFA levels and Z-scaled log-transformed postprandial lipemic and glycemic parameters running linear mixed models adjusting for age, BMI and family relatedness as random effects.

#### Host genetics contribution to SCFA levels: heritability estimates

To estimate the heritability of the SCFA levels in serum (fasting and postprandial) and stool, we utilized the classical twin model and compared the degree of similarity among monozygotic (MZ) twins, who share 100% of their genetic make-up, and dizygotic (DZ) twins, who share on average 50% of their segregating genes. Under the equal environment assumption (EEA), the variance of the trait/phenotype (P) is explained by three latent parameters: additive genetic variance (A), shared (familial) environmental variance (C) and individual-specific environmental variance/error (E).^39^ To estimate the heritability, we utilized the structural equation models (SEM), which uses the observed covariances from both MZ and DZ pairs to establish a causal relationship between the covariances and the latent parameters. We performed the heritability analysis using the twinlm function (R METs package).^40^ Heritability of the SCFA levels in fasting serum and stool was calculated in TwinsUK and ZOE PREDICT- 1 participants together to increase the sample size ensuring accurate estimates. Heritability models of the fecal and fasting circulating SCFA levels were adjusted for age, sex and BMI, whereas models of the postprandial levels were adjusted for age and BMI (sex was not included as all the participants were women).

##### Gut microbiota contribution to SCFA levels: random forest models

The machine learning framework employed is based on the scikit-learn Python package.^41^ The ML algorithms used for the prediction of SCFAs in serum (fasting and postprandial) and stool from the species-level relative abundances (as estimated by MetaPhlAn 4.beta.2 and normalized using the arcsin-sqrt transformation for compositional data) are based on Random Forest (RF) classification and regression. We selected RF-based methods a priori as it has been repeatedly shown to be particularly suitable and robust to the statistical challenges inherent to microbiome abundance data.^42^ A cross-validation approach was implemented, based on 100 bootstrap iterations and an 80/20 random split into training and testing folds. To specifically avoid overfitting due to the twin nature of our data and their shared factors, we removed any twin from the training fold if their twin was present in the test fold.

For the classifiers, we divided the continuous features into two classes: the top and bottom quartiles. From the scikit-learn package, we used the RandomForestClassifier function with n_estimators = 1000, max_features=‘sqrt’ parameters. For the regressors, we trained an RF regressor to learn the feature to predict and simple linear regression to calibrate the output for the test folds on the range of values in the training folds. From the scikit-learn package, we used the RandomForestRegressor function with n_estimators = 1000, criterion=’mse’, max_features=‘sqrt’ parameters and LinearRegression with default parameters.

As an additional control, we verified that when randomly swapping the target labels or values (classification and regression, respectively), the performances were reflecting a random prediction, hence an area under the ROC curve (AUC) very close to 0.5 and a non-significant correlation between the real and predicted values approaching 0.

###### Links between circulating SCFA levels and chronic and acute inflammatory responses, and differences in SCFA levels between controls and acute fracture patients

Circulating SCFA levels and cytokines were log-transformed to obtain a normal distribution and then Z-scaled. We first assessed the associations between SCFA levels and cytokines in healthy individuals from the acute trauma case-control study, a subset of TwinsUK with measurements of circulating cytokines and SCFAs, and ZOE PREDICT-1 by running Pearson’s correlations. We then combined the results from the different studies using an inverse variance random effect meta-analysis. Moreover, Pearson’s correlations between each marker (IL-10, TNF-α, IFN-γ GlycA, IL-6) and SCFA stratifying by the type of acute trauma (hip fracture or rib fracture) were also run to investigate the potential link between SCFA levels and acute inflammatory responses. Pairwise t.test and linear models were employed to test differences in circulating SCFAs between trauma patients (hip/rib fracture) and controls. Linear models were further adjusted for age (set as a two levels factor defined by age ≥ 50 and age < 50) and sex.

## Supplementary Material

Supplemental Material
