# Supplementary material for: Genetic and gut microbiome determinants of SCFA circulating and fecal levels, postprandial responses and links to chronic and acute inflammation
Source: Gut Microbes. 2023 Aug 1;15(1):2240050. doi: 10.1080/19490976.2023.2240050 (PMC10395212; doi:10.1080/19490976.2023.2240050)
Supplement: Supplemental Material [file KGMI_A_2240050_SM1400.docx]

Genetic and gut microbiome determinants of SCFA circulating and faecal levels, postprandial responses and links to acute inflammation

Ana Nogal^1^, Francesco Asnicar^2^, Amrita Vijay^3^, Afroditi Kouraki^3^, Alessia Visconti^1^, Panayiotis Louca^1^, Kari Wong^4^, Andrei-Florin Baleanu^1^, Francesca Giordano^5^, Jonathan Wolf^5^, George Hadjigeorgiou^5^, Richard Davies^5^, Gregory A. Michelotti^4^, Paul W. Franks^6,7^, Sarah E. Berry^8^, Mario Falchi^1^ Adeel Ikram^3^, Benjamin J. Ollivere^3^, Amy Zheng^3^, Jessica Nightingale^3^, Massimo Mangino^1,9^, Nicola Segata^2^, William J. Bulsiewicz^5^, Tim D Spector^1^, Ana M Valdes^3^*, Cristina Menni^1^*

**Supplementary Material**

[**Supplementary Figure 1**. Spearman’s correlations between age and BMI, and SCFAs in serum and stool in participants from the TwinsUK and ZOE PREDICT-1 cohorts. 2](#_Toc127789219)

[**Supplementary Figure 2**. Partial Spearman’s correlations between abundances of single gut microbial species and faecal SCFAs levels for 1178 individuals from TwinsUK and ZOE PREDICT-1. 3](#_Toc127789220)

[**Supplementary Table 1**. Postprandial changes from fasting and inter-individual variability for each SCFA assessed using Wilcoxon tests and coefficient of variation (CV- calculated as SD/mean, %). 4](#_Toc127789221)

[**Supplementary Table 2.** Associations between postprandial SCFA levels and postprandial lipaemic and glycaemic parameters in ZOE PREDICT-1 participants. 5](#_Toc127789222)

[**Supplementary Table 3.** Influence of the gut microbiota composition in faecal and circulating SCFA levels estimated by Random Forest regression (using Spearman’s correlations) and classification (using AUC) models. 6](#_Toc127789223)

[**Supplementary Table 4.** Demographic characteristics of the participants from the subset of TwinsUK with measurements of circulating SCFAs and cytokines, and the acute trauma case-control cohort. 8](#_Toc127789224)

[**Supplementary Table 5.** Associations between circulating SCFA levels and fracture in individuals from the acute trauma case-control cohort. 9](#_Toc127789225)

[**Supplementary Text 1.** Full details and quality control of the SCFA measurements. 10](#_Toc127789226)


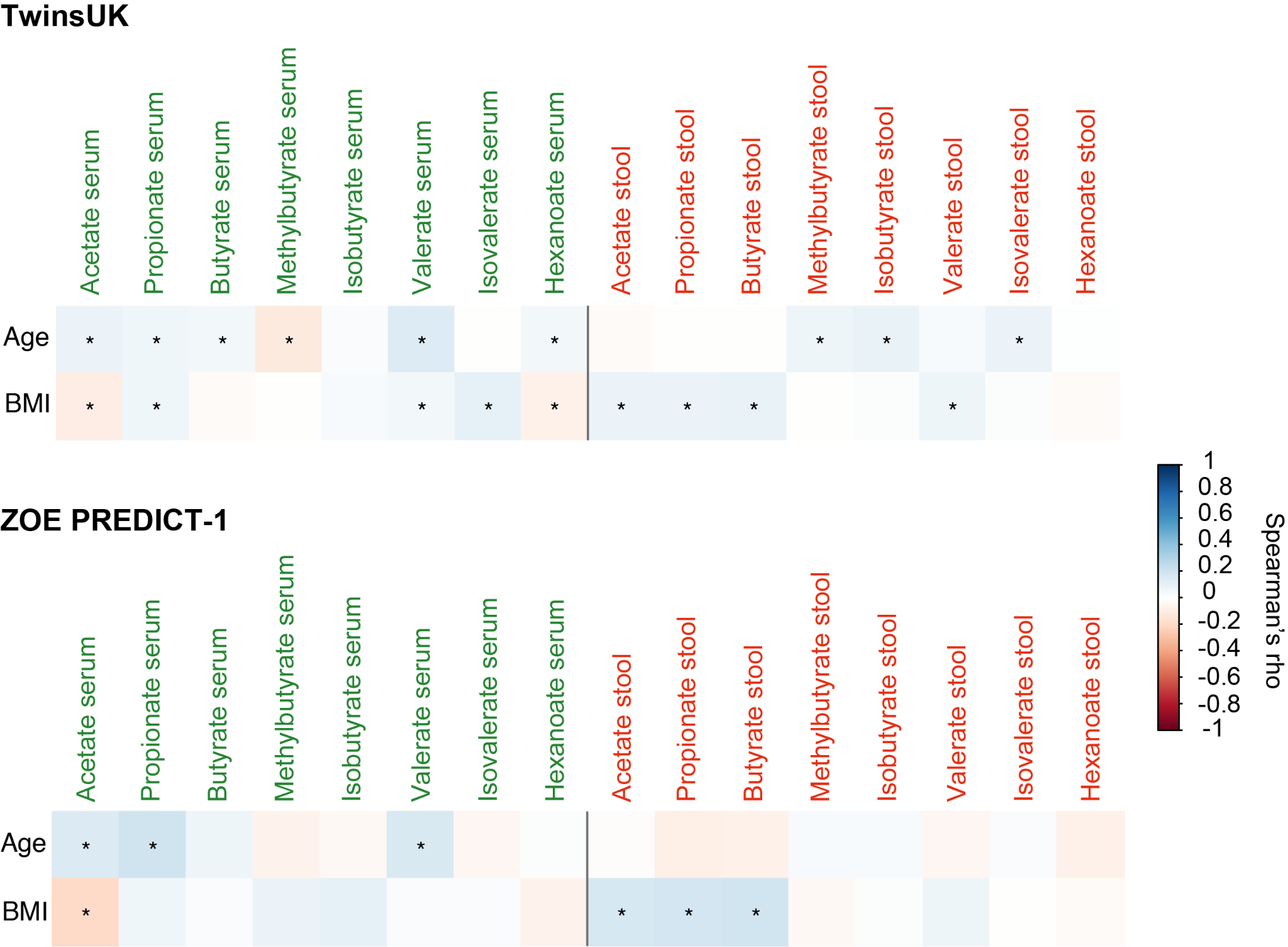


## **Supplementary Figure 1**. Spearman’s correlations between age and BMI, and SCFAs in serum and stool in participants from the TwinsUK and ZOE PREDICT-1 cohorts. Significant correlations (FDR <0.05) are indicated with an asterix.

##


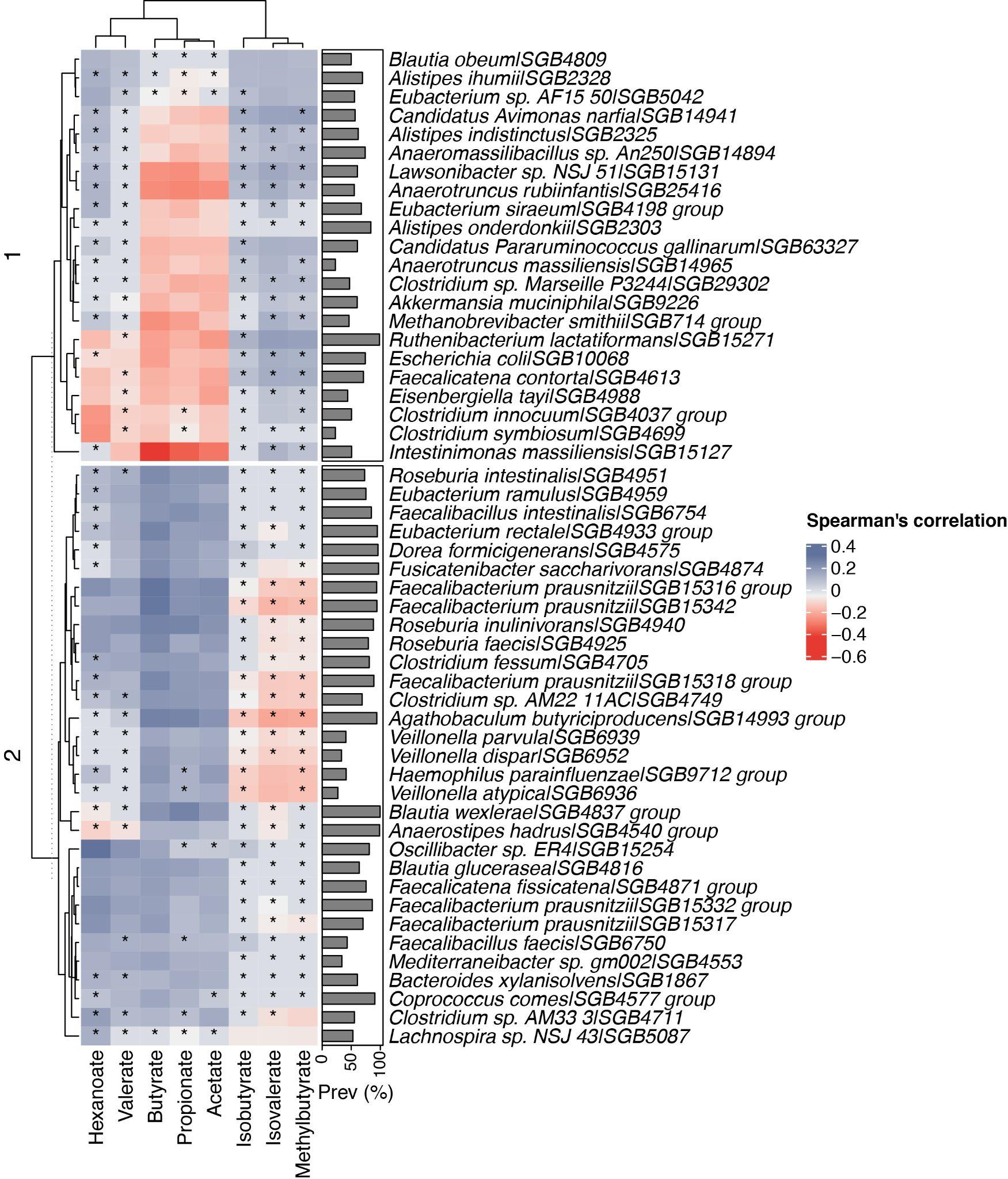


## **Supplementary Figure 2**. Partial Spearman’s correlations between abundances of single gut microbial species and faecal SCFAs levels for 1178 individuals from TwinsUK and ZOE PREDICT-1. Correlations were adjusted for age, BMI and sex. Characterised species with a prevalence>20%, presenting significant correlations in the 3 datasets - TwinsUK together with ZOE PREDICT-1 (FDR<0.2), TwinsUK, and ZOE PREDICT-1 (nominal p-value=0.05) -, and with at least 3 different SCFAs were presented. Correlations that were not replicated in TwinsUK and/or ZOE PREDICT-1 (nominal p-value≥0.05) are indicated with an asterisk. The species are presented using their Species-level Genome Bins (SGBs) identifiers. The species and SCFAs were hierarchically clustered (complete linkage, Euclidean distance). The horizontal bars indicate the prevalence values (%) for each species.

## **Supplementary Table 1**. Postprandial changes from fasting and inter-individual variability for each SCFA assessed using Wilcoxon tests and coefficient of variation (CV- calculated as SD/mean, %).

| **SCFA** | **Postprandial measure** | **CV** | **Wilcoxon p-value** |
| --- | --- | --- | --- |
| Acetate | Peak | 36.8 | 9.6 x 10^-5^ |
|  | Dip | 41.6 | 4.1 x 10^-52^ |
| Propionate | Peak | 28.2 | 3.6 x 10^-23^ |
|  | Dip | 37.9 | 1.3 x 10^-11^ |
| Butyrate | Peak | 33.6 | 4.0 x 10^-28^ |
|  | Dip | 41.3 | 6.1 x 10^-11^ |
| Methylbutyrate | Peak | 29.8 | 0.37 |
|  | Dip | 36.5 | 1.5 x 10^-35^ |
| Isobutyrate | Peak | 26.5 | 9.6 x 10^-5^ |
|  | Dip | 32 | 6.9 x 10^-40^ |
| Valerate | Peak | 32.7 | 5.9 x 10^-32^ |
|  | Dip | 46.7 | 0.08 |
| Isovalerate | Peak | 25.4 | 1.4 x 10^-17^ |
|  | Dip | 33.8 | 1.2 x 10^-18^ |
| Hexanoate | Peak | 39.8 | 0.37 |
|  | Dip | 39.2 | 2.1 x 10^-27^ |

## **Supplementary Table 2.** Associations between postprandial SCFA levels and postprandial lipaemic and glycaemic parameters in ZOE PREDICT-1 participants. The beta estimates and p-values (FDR) (shown in parenthesis) obtained from linear mixed models adjusted for age, BMI and family relatedness are reported.

| **SCFA** | **Postprandial measure** | **Triglycerides 6h-rise** | **Glucose iAUC0-2h** | **C-peptide 2h-rise** | **Insulin 2h-rise** |
| --- | --- | --- | --- | --- | --- |
| Acetate | Peak | 0.15 (0.11) | 0.04 (0.91) | 0.02 (0.95) | 0.08 (0.68) |
|  | Dip | 0.22 (0.001) | -0.2 (0.005) | -0.01 (0.97) | 0.06 (0.64) |
| Propionate | Peak | 0.01 (0.95) | 0.15 (0.11) | 0.08 (0.7) | 0.04 (0.91) |
|  | Dip | 0.06 (0.63) | 0.15 (0.07) | 0.12 (0.13) | 0.07 (0.54) |
| Butyrate | Peak | -0.06 (0.91) | 0.14 (0.11) | 0.06 (0.91) | 0.04 (0.91) |
|  | Dip | 0.03 (0.93) | 0.14 (0.08) | 0.12 (0.11) | 0.07 (0.55) |
| Methylbuty-rate | Peak | 0.06 (0.91) | 0.04 (0.91) | 0.05 (0.91) | 0.04 (0.91) |
|  | Dip | 0.02 (0.94) | -0.08 (0.5) | -0.03 (0.93) | -0.03 (0.93) |
| Isobutyrate | Peak | 0.03 (0.91) | -0.01 (0.97) | 0 (0.97) | 0 (0.97) |
|  | Dip | 0.01 (0.98) | -0.07 (0.54) | 0 (0.98) | -0.02 (0.94) |
| Valerate | Peak | -0.03 (0.91) | 0.14 (0.14) | 0.02 (0.95) | 0.05 (0.91) |
|  | Dip | -0.03 (0.93) | 0.14 (0.08) | 0.03 (0.93) | 0.02 (0.94) |
| Isovalerate | Peak | -0.02 (0.95) | -0.03 (0.91) | 0.02 (0.95) | 0 (0.97) |
|  | Dip | 0 (0.98) | -0.16 (0.04) | -0.02 (0.94) | -0.01 (0.94) |
| Hexanoate | Peak | -0.03 (0.95) | 0.07 (0.83) | 0.01 (0.95) | -0.01 (0.97) |
|  | Dip | -0.03 (0.93) | 0.05 (0.69) | 0 (0.98) | -0.04 (0.81) |

## **Supplementary Table 3.** Influence of the gut microbiota composition in faecal and circulating SCFA levels estimated by Random Forest regression (using Spearman’s correlations) and classification (using AUC) models. The median AUC and the 95% confidence intervals across 100 folds for a corresponding binary classifier between the highest and lowest quartile, and the median values and the 95% confidence intervals of the Spearman’s correlation between the real value of each component and the value predicted by regression models across 100 training/testing folds are shown.

| **Sample** | **SCFA** | **Cohort** | **AUC** | **AUC 95% CI** | **Spearman’s rho** | **Spearman’s rho 95% CI** |
| --- | --- | --- | --- | --- | --- | --- |
| Serum | Acetate | TwinsUK | 0.51 | 0.51,0.53 | 0.07 | 0.06,0.09 |
|  |  | ZOE PREDICT-1 | 0.57 | 0.56,0.59 | 0.05 | 0.02,0.07 |
|  | Propionate | TwinsUK | 0.51 | 0.51,0.53 | 0.03 | 0,0.03 |
|  |  | ZOE PREDICT-1 | 0.67 | 0.65,0.68 | 0.17 | 0.15,0.19 |
|  | Butyrate | TwinsUK | 0.6 | 0.59,0.61 | 0.06 | 0.04,0.07 |
|  |  | ZOE PREDICT-1 | 0.61 | 0.6,0.63 | 0.12 | 0.09,0.14 |
|  | Methylbuty-rate | TwinsUK | 0.6 | 0.59,0.61 | 0.1 | 0.08,0.11 |
|  |  | ZOE PREDICT-1 | 0.52 | 0.5,0.54 | 0 | -0.01,0.03 |
|  | Isobutyrate | TwinsUK | 0.56 | 0.56,0.58 | 0.08 | 0.07,0.09 |
|  |  | ZOE PREDICT-1 | 0.52 | 0.5,0.53 | 0.01 | 0,0.04 |
|  | Valerate | TwinsUK | 0.61 | 0.61,0.63 | 0.14 | 0.13,0.16 |
|  |  | ZOE PREDICT-1 | 0.59 | 0.57,0.6 | 0.08 | 0.05,0.1 |
|  | Isovalerate | TwinsUK | 0.5 | 0.48,0.51 | -0.02 | -0.04,-0.01 |
|  |  | ZOE PREDICT-1 | 0.57 | 0.54,0.58 | 0.05 | 0.04,0.08 |
|  | Hexanoate | TwinsUK | 0.63 | 0.62,0.64 | 0.19 | 0.16,0.19 |
|  |  | ZOE PREDICT-1 | 0.56 | 0.53,0.57 | 0.02 | -0.01,0.04 |

| Stool | Acetate | TwinsUK | 0.82 | 0.81,0.82 | 0.43 | 0.41,0.43 |
| --- | --- | --- | --- | --- | --- | --- |
|  |  | ZOE PREDICT-1 | 0.91 | 0.9,0.92 | 0.59 | 0.56,0.59 |
|  | Propionate | TwinsUK | 0.82 | 0.81,0.82 | 0.47 | 0.45,0.48 |
|  |  | ZOE PREDICT-1 | 0.93 | 0.91,0.93 | 0.62 | 0.6,0.63 |
|  | Butyrate | TwinsUK | 0.86 | 0.85,0.86 | 0.55 | 0.54,0.55 |
|  |  | ZOE PREDICT-1 | 0.91 | 0.89,091 | 0.61 | 0.59,0.62 |
|  | Methylbuty-rate | TwinsUK | 0.78 | 0.77,0.79 | 0.41 | 0.39,0.42 |
|  |  | ZOE PREDICT-1 | 0.64 | 0.62,0.66 | 0.19 | 0.15,0.2 |
|  | Isobutyrate | TwinsUK | 0.75 | 0.74,0.76 | 0.33 | 0.31,0.34 |
|  |  | ZOE PREDICT-1 | 0.6 | 0.58,0.62 | 0.1 | 0.08,0.13 |
|  | Valerate | TwinsUK | 0.75 | 0.73,0.75 | 0.35 | 0.34,0.36 |
|  |  | ZOE PREDICT-1 | 0.78 | 0.75,0.78 | 0.33 | 0.31,0.35 |
|  | Isovalerate | TwinsUK | 0.78 | 0.78,0.79 | 0.42 | 0.4,0.43 |
|  |  | ZOE PREDICT-1 | 0.66 | 0.64,0.67 | 0.23 | 0.2,0.25 |
|  | Hexanoate | TwinsUK | 0.83 | 0.83,0.84 | 0.46 | 0.45,0.47 |
|  |  | ZOE PREDICT-1 | 0.82 | 0.8,0.83 | 0.39 | 0.37,0.42 |

## **Supplementary Table 4.** Demographic characteristics of the participants from the subset of TwinsUK with measurements of circulating SCFAs and cytokines, and the acute trauma case-control cohort.

| **Cohort** | **Type** | **n** | **Females, (%)** | **Age, yrs** |
| --- | --- | --- | --- | --- |
| Acute trauma case-control | Healthy (controls) | 21 | 55% | 38.7 (14.97) |
|  | Rib fracture | 18 | 38% | 59.6 (16.18) |
|  | Hip fracture | 32 | 80% | 88.7 (5.03) |
| TwinsUK | Healthy | 82 | 100% | 67.6 (10.9) |

## **Supplementary Table 5.** Associations between circulating SCFA levels and fracture in individuals from the acute trauma case-control cohort. Results are presented without adjusting and after adjusting for age and sex.

|  |  | *Without adjusting* | | | *Adjusting for age and sex* | | |
| --- | --- | --- | --- | --- | --- | --- | --- |
| SCFA | **Compared groups** | **Beta** | **SE** | **P-value** | **Beta** | **SE** | **P-value** |
| Acetate | Hip-Control | 1,51 | 0,23 | 0 | 1,82 | 0,36 | 0 |
|  | Rib-Control | 0,39 | 0,26 | 0,14 | 0,61 | 0,32 | 0,06 |
|  | Rib-Hip | -1,11 | 0,23 | 0 | -1,21 | 0,26 | 0 |
| Propionate | Hip-Control | 0,75 | 0,28 | 0,01 | 1,11 | 0,43 | 0,01 |
|  | Rib-Control | 0,79 | 0,32 | 0,02 | 1,13 | 0,39 | 0 |
|  | Rib-Hip | 0,04 | 0,28 | 0,88 | 0,03 | 0,32 | 0,93 |
| Butyrate | Hip-Control | 0,07 | 0,31 | 0,83 | 0,34 | 0,46 | 0,47 |
|  | Rib-Control | 0,13 | 0,34 | 0,71 | 0,44 | 0,41 | 0,29 |
|  | Rib-Hip | 0,06 | 0,31 | 0,84 | 0,1 | 0,34 | 0,76 |
| Methylbutyrate | Hip-Control | -0,52 | 0,28 | 0,07 | -0,51 | 0,43 | 0,24 |
|  | Rib-Control | 0,48 | 0,31 | 0,13 | 0,46 | 0,38 | 0,24 |
|  | Rib-Hip | 1 | 0,28 | 0 | 0,97 | 0,32 | 0 |
| Isobutyrate | Hip-Control | 0,12 | 0,3 | 0,69 | 0,19 | 0,47 | 0,69 |
|  | Rib-Control | 0,2 | 0,34 | 0,55 | 0,15 | 0,42 | 0,71 |
|  | Rib-Hip | 0,08 | 0,3 | 0,79 | -0,03 | 0,35 | 0,92 |
| Valerate | Hip-Control | 0,63 | 0,3 | 0,04 | 1,14 | 0,44 | 0,01 |
|  | Rib-Control | 0,36 | 0,33 | 0,29 | 0,82 | 0,4 | 0,04 |
|  | Rib-Hip | -0,27 | 0,3 | 0,36 | -0,32 | 0,33 | 0,33 |
| Isovalerate | Hip-Control | 0,66 | 0,28 | 0,02 | 0,84 | 0,44 | 0,06 |
|  | Rib-Control | 0,95 | 0,32 | 0 | 1 | 0,39 | 0,01 |
|  | Rib-Hip | 0,28 | 0,28 | 0,32 | 0,16 | 0,32 | 0,63 |
| Hexanoate | Hip-Control | 0,02 | 0,3 | 0,96 | 0,19 | 0,47 | 0,69 |
|  | Rib-Control | 0,08 | 0,34 | 0,82 | 0,14 | 0,42 | 0,75 |
|  | Rib-Hip | 0,06 | 0,3 | 0,84 | -0,05 | 0,35 | 0,88 |

## **Supplementary Text 1.** Full details and quality control of the SCFA measurements.

Human serum and stool samples were spiked with stable labelled internal standards, homogenized and subjected to protein precipitation with an organic solvent. After centrifugation, an aliquot of the supernatant is derivatized. The reaction mixture was injected onto an Agilent 1290/AB Sciex QTrap 5500 LC MS/MS system equipped with a C18 reversed phase UHPLC column. The mass spectrometer is operated in negative mode using electrospray ionization (ESI). The peak area of the individual analyte product ions was measured against the peak area of the product ions of the corresponding internal standards. Quantitation was performed using a weighted linear least squares regression analysis generated from fortified calibration standards prepared immediately prior to each run. LC-MS/MS raw data were collected and processed using AB SCIEX software Analyst 1.6.3 and processed using SCIEX OS-MQ software v1.7

Sample analyses were carried out in a 96-well plate format containing two calibration curves. Accuracy was evaluated using the corresponding QC replicates in the sample runs. QCs met acceptance criteria at all levels for all analytes (QC acceptance criteria: At least 50% of QC samples at each concentration level per analyte should be within ±20.0% of the corresponding historical mean, and at least 2/3 of all QC samples per analyte should fall within ±20.0% of the corresponding historical mean).
